# Systematic Review of Effects of Medication Dispenser Use by Home-Dwelling Older Adults

**DOI:** 10.1097/NNR.0000000000000824

**Published:** 2025-04-02

**Authors:** Olli Salmensuu, Virva Hyttinen-Huotari, Jenni Isotalo, Mieke Rijken, Ismo Linnosmaa, Minna Kaarakainen

**Affiliations:** **Olli Salmensuu, PhD, is Postdoctoral Researcher**, University of Eastern Finland, Kuopio, Finland.; **Virva Hyttinen-Huotari, PhD, is Assistant Professor**, University of Eastern Finland, Kuopio, Finland.; **Jenni Isotalo, MSc, RN, is Nurse**, Wellbeing Services County of South Ostrobothnia, Seinäjoki, Finland.; **Mieke Rijken, PhD, is Professor**, University of Eastern Finland, Kuopio, Finland and Senior researcher, Nivel, Utrecht, the Netherlands.; **Ismo Linnosmaa, PhD, is Professor**, University of Eastern Finland, Kuopio, Finland and Research professor, THL, Helsinki, Finland.; **Minna Kaarakainen, PhD, RN, is Visiting Researcher**, University of Eastern Finland, Kuopio, Finland.

**Keywords:** independent living, medication systems, treatment outcome

## Abstract

**Background:**

Population aging has increased the need for solutions that help older adults live independently in their own homes, where medication management is a major challenge.

**Objectives:**

In this systematic review, we assessed the effects of medication dispensers among home-dwelling older adults on outcomes within the five domains of the Quintuple Aim framework: user experiences, health and well-being outcomes, health service utilization and costs, care professional experiences, and equity.

**Methods:**

We identified relevant studies by searching databases (Scopus, CENTRAL, PubMed, Web of Science, CINAHL, PsycINFO, and Cochrane Reviews) from January 2017 to April 2022 with a predefined search strategy and two-person abstract and full-text screening. Two authors extracted the most relevant data and assessed quality for each included study. We assessed the evidence using a four-level quality rating measure: strong, moderate, limited, or no evidence.

**Results:**

We included five original studies and three systematic reviews, which provided information on 20 additional original studies. Data were extracted from these 25 original studies. We found significant results in 16 of them, mostly pointing to the beneficial effects of dispenser devices. Significant results for health and well-being outcomes were found in 13 out of 21 studies in which these were assessed, for service utilization in two out of five studies, for costs in two out of three studies, and for patient/carer experiences in one out of five studies. No study evaluated professional experiences or equity outcomes. Overall, strong evidence of a beneficial effect of dispenser devices in any outcome is lacking, but they can improve health outcomes (moderate evidence of beneficial effects of using dispenser devices on systolic and diastolic blood pressure, and hemoglobin A1c levels). For other outcome domains, there is no or only limited evidence for beneficial effects of dispenser devices.

**Discussion:**

We found that the use of dispenser devices by home-dwelling older adults can improve clinical health outcomes and may reduce health service utilization and costs. More high-quality research is needed to get a better insight into their effects on service utilization and costs. Future studies should also examine the effects on care professionals’ experiences and equity.

Globally, aging populations have increased the demand for innovative solutions aimed at facilitating independent living in a home for as long as possible, which most older adults also desire (Roy et al., 2018). A challenge arises from the fact that the prevalence of illnesses increases with age, thereby raising the risk of polypharmacy. This leads to a complex regimen that many older adults follow (Bell et al., 2021). Medication management stands out as one of the major challenges in independent living of older adults (Maher et al., 2014). Personal medication management devices (PMMDs) have been promising in improving medication adherence and preventing medication errors (Turjamaa et al., 2020; Watson et al., 2016). Additionally, such technology can free up health care personnel’s time for other tasks (Turjamaa et al., 2020); however, little is known about the experiences of patients or health care professionals regarding the changes in everyday routines after the implementation of a medication dispenser (Mertz et al., 2021). Although medication dispensers have been previously evaluated regarding their effects on medication adherence (e.g., Cross et al., 2020; Kurup et al., 2020), more research is needed on their effects on health and well-being outcomes, costs and cost-effectiveness, and especially on how they support independent living of older adults (Khosravi and Ghapanchi, 2016; Turjamaa et al., 2020; Watson et al., 2016).

With this paper, we aim to answer the following question: What are the effects of using medication dispensers by home-dwelling older adults on outcomes within the five domains of the Quintuple Aim for health care improvement framework (Nundy et al., 2022): (a) care experiences of users, i.e., older adults and family/caregivers; (b) well-being and experiences of care professionals (e.g., job satisfaction, workload); (c) health and well-being outcomes; (d) use of health and social care/costs/cost-effectiveness; and (e) equity. Extending the original Triple Aim framework (Berwick et al., 2008), it simultaneously addresses the five domains, thus setting up a comprehensive foundation for designing policies that optimize health and care systems that are not only effective and efficient but also equitable and sustainable. We study the effects of medication dispensers from their wider scope to gather evidence on how such devices can contribute to cost containment without adverse effects on older adults’ health and well-being, thus supporting financial sustainability of health and social care systems in the era of aging populations. Also, the equity perspective is important as not all older adults benefit equally from the devices.

The review focused on the effects of medication dispensers on outcomes within the five Quintuple Aim domains—and not on studies that evaluated multicomponent interventions, of which dispensers were only one of multiple intervention elements—as it would be impossible to filter out the effects of the other intervention elements on the outcomes of interest. Also, we did not evaluate intermediate outcomes such as changes in users’ medication knowledge or behavior (e.g., medication adherence) but focused on outcomes particularly relevant to service design and policymaking. To study the benefits for older users but not limit the number of studies too much, we included studies in which at least part of the study sample was 65 years or older. Regarding the user experiences, we were not interested in user satisfaction related to the attractiveness, usability, or feasibility of medication dispensers but in changes in the experiences of users, their caregivers, and care professionals that are related to their care.

## METHODS

The literature on PMMD interventions has interwoven dispensing and reminding functions of the devices, which necessitates that systematic searches encompass both functions as the true nature of the intervention can often be decided only at the full-text phase of the screening. Therefore, to allow retracing false rejections related to the intervention, we parallel screened dispensing and reminder interventions in both the title and abstract screening and full-text screening phases of the review. In this systematic review, we will report on the effects of medication dispensers, which are devices used to store and dispense medication and, contrarily to Watson et al. (2016), may also have reminder elements. Our approach is more comprehensive than what is usually encountered in systematic reviews. In addition to randomized controlled trials (RCTs), we synthesized information from systematic reviews. By extracting data from these systematic reviews at the level of the included original studies (which met our inclusion and exclusion criteria), we were able to synthesize more precisely the results of the included reviews that were relevant to our systematic review and their bias assessments. We complemented external assessments of result bias by assessing the completeness of their summaries using our criteria.

### Search Strategy and Data Sources

Helped by a librarian experienced in literature reviews, relevant databases were selected, and a search strategy was developed, tested, and further refined. As a result, we searched for relevant articles from PubMed, Cochrane (CENTRAL and Reviews), Scopus, WoS, CINAHL, and PsycINFO using the following search terms: (effect* OR cost* OR financ* OR pric* OR adher*) AND (remind* OR dispens*) AND (sms OR smartphone OR tablet* OR automat* OR robot* OR tech* OR electr* OR device OR digital OR application* OR manag*) AND (medic* OR drug* OR pharma*) AND (home OR “assisted living” OR “independent living” OR ehealth OR mhealth OR “informal care*” OR nonhospital OR “self-manage*”) AND (aging OR ageing OR senior* OR elderly OR polypharmacy OR multimorbidity) except for Scopus for which the first AND was replaced by W/1000 and all the other ANDs by W/100. It should be noted that our search terms were broader to capture evaluation studies on PMMDs more generally than other existing reviews. In addition, separating interweaved reminding and dispensing functions of the interventions also needed full-text screening. Cochrane protocols, editorials, special collections, and clinical answers were filtered out. We conducted all the searches from January 1, 2017 to April 11, 2022, noting that results of older original articles would still be accounted for as part of any included systematic reviews published during the chosen period. No language restrictions were made.

### Study Selection

**FIGURE 1:**
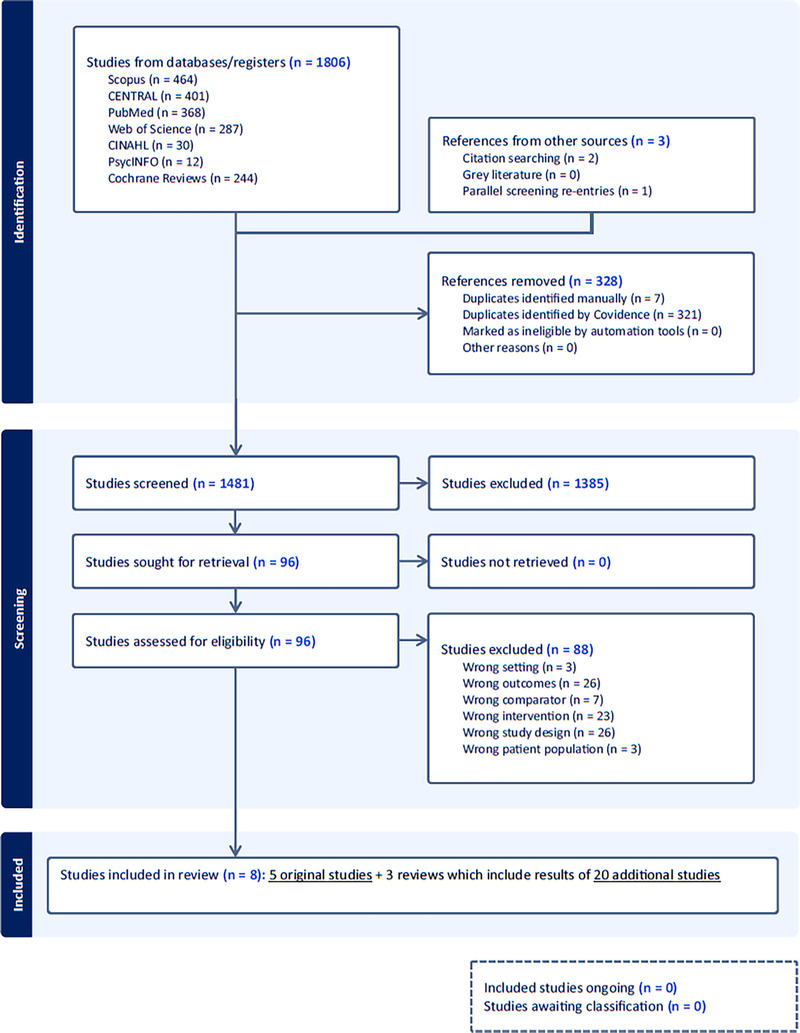
PRISMA flow diagram on study selection. *Notes.* CENTRAL = Cochrane Central Register of Controlled Trials; CINAHL = Cumulated Index to Nursing and Allied Health Literature.

The inclusion and exclusion criteria are found in Table 1. Supplemental Digital Content (SDC) Table 1 (http://links.lww.com/NRES/A561) presents the completed Preferred Reporting Items for Systematic Reviews and Meta-Analyses (PRISMA) checklist. Figure 1 depicts the PRISMA flow diagram of the study selection.

**TABLE 1:** Inclusion and Exclusion Criteria

Inclusion criteria	Exclusion criteria
(Participants) Study participants include people aged ≥65 years living in their own home. (Interventions) Study focuses on, or a substantial part of the intervention consists of, using a medication dispenser device. (Comparators) Study is an experimental or observational study including a comparative element (e.g., data collected from a control group, data collected prior to the implementation of the intervention, reference data) or the study is a systematic review that includes at least one original study passing all inclusion criteria.	(Outcomes) Study does not analyze or synthesize effects on outcomes within at least one of the Quintuple Aim domains: 1) Care experiences of older people and caregivers; 2) Well-being and experiences of care professionals (e.g., workload, job satisfaction); 3) Health and/or well-being outcomes; 4) Use of health and/or social care/costs/cost-effectiveness; 5) Equity.

Database searches yielded a total of 1,806 records. All the retrieved records were transferred into Covidence software (https://www.covidence.org/). Duplicates were removed both automatically and manually. Using the predetermined inclusion and exclusion criteria, two reviewers (OS and VHH) screened the titles and abstracts to identify potentially eligible articles for inclusion. Full texts that passed the initial screening phase were screened by two reviewers (OS and VHH) using the same criteria. The two reviewers discussed ambiguities concerning the inclusion of the articles and, where needed, also by the wider research team to reach consensus. Full-text screening of 96 articles resulted in five original studies and three systematic reviews to be included in this review (SDC Table 2 [http://links.lww.com/NRES/A561] lists the 88 studies that were excluded at this stage with reasons). The studies included in the systematic reviews were manually searched for original research that met the inclusion and exclusion criteria and had not been identified yet. This resulted in adding 20 original studies with publication dates before January 1, 2017.

Furthermore, we hand-searched two original studies not found by the applied strategy (one to replace its process evaluation sister article whereas the other was found when searching for a published full text of an abstract), but they did not pass the criteria for inclusion. The hand-searched studies were similarly assessed by two reviewers (OS and JI). This approach resulted in a total of 25 original studies included in this review.

### Data Extraction and Quality Assessment

Two reviewers (OS and JI) extracted data from the 25 original studies. The most critical information was extracted for each included study by the two reviewers separately and subsequently discussed. In addition to these more critical data that were extracted by the two reviewers, some general information was extracted by one reviewer (OS). Two reviewers (OS and JI) assessed the risk of bias for each study, discussing with the research team where ambiguities arose. For the three systematic reviews, the complementary assessment followed “A critical appraisal tool for systematic reviews that include randomized or nonrandomized studies of healthcare interventions, or both” (AMSTAR2; Shea et al., 2017). For the initially identified five original studies, we used the “Mixed Methods Appraisal Tool” (MMAT; Hong et al., 2018). An institutional review board statement is not applicable as the systematic review procedure extracts, analyses, and synthesizes existing data.

### Evidence Levels

To draw conclusions on the effectiveness, we used four levels of evidence:

1. Strong evidence: consistent evidence for a beneficial (i.e., desired direction benefiting health, relief from burden, cost savings) and significant effect of dispensers in an outcome measure across multiple (at least three) RCT studies with high-quality scores (4 or 5) or (for studies found in systematic reviews) similar ranking.
2. Moderate evidence: Consistent evidence for a beneficial effect across multiple studies, including at least one significant finding in an RCT study with a high-quality score (4 or 5) or similar ranking.
3. Limited evidence: Consistent findings—with at least one significant finding—for a beneficial effect of a dispenser device in an outcome measure.
4. No evidence: Nonsignificant or inconsistent evidence across multiple studies. Results were considered consistent, where at least 75% of the studies showed results in the same direction supporting the intervention (such as dispensers enhancing health or well-being or reducing costs). For significant evidence, similar consistency was required.

## RESULTS

### Study and Intervention Characteristics

To this review, we included five original studies (listed in Table 2) and three systematic reviews (Table 3) from which we retrieved results of 20 additional original studies; thus, in total, we have results of 25 original studies. Study and intervention characteristics together with all outcome results are described with more detail in SDC Tables 3 and 4 (http://links.lww.com/NRES/A561). Dispenser solutions in our study included self-managed dispenser devices, predispensed packages, and automated home dispensing technologies. By continents, 18 studies were conducted in the United States and Canada, 3 in Europe, 3 in Australia and New Zealand, and 1 in Asia. Research has been carried out and published only in high-income countries, taking into consideration that the Asian study is from Japan. Dispenser devices were mostly used by older adults. In 14 studies, user mean age ranged from 64 to 80 years, whereas in four studies, mean age was below 50 years. By study types, we found 11 RCTs, 1 case series study, 1 cross-sectional study, 2 cohort studies, and 10 non-RCTs (also reported as controlled clinical trials or quasi-RCTs in overlapping reviews). Intervention lengths ranged from 10 days to 14 months.

**TABLE 2:** Results of Methodological Quality Assessment: Medication Dispenser Original Studies

Study	Randomization/representativeness	Similar at baseline/appropriate measurements	Complete outcome data/low nonresponse bias risk	Blinding of assessors/confounding accounted for	Adherence to intervention/exposure as intended/strategy	Total
Arain et al. (2021)	1	0	0	0	0	1/5
Hoffmann et al. (2018)	0	1	1	0	1	3/5
Kamimura (2019)	0	1	1	0	0	2/5
Mertens et al. (2020)	1	0	1	0	1	3/5
Polenick et al. (2020)	1	1	1	1	1	5/5

*Note.* MMAT questions 1–5 by study type: RCTs: Arain et al. (2021), Mertens et al. (2020); Quantitative nonrandomized studies: Hoffmann et al. (2018), Polenick et al. (2020); Quantitative descriptive study: Kamimura (2019)—note for the descriptive study: the MMAT question order is changed here so that it begins with the second question “Is the sample representative of the target population?” and the first question on relevant sampling strategy is in the fifth place.

**TABLE 3:** Results of Quality Assessment: Medication Dispenser Systematic Reviews

	Cross et al. (2020)	Singh & Varshney (2019)	Stevenson et al. (2019)
PICO components	1	0	1
Protocol or prior methods	1	0	1
RCT/NRSI selection explained	0	0	0
Comprehensive search strategy	1	½	1
Duplicate study selection	1	0	1
Duplicate data extraction	1	0	1
List of excluded studies	1	0	0
Studies described in detail	1	0	1
Satisfactory RoB technique	1	0	1
Reported on funding of studies	1	0	1
MA: appropr. stat. methods	1	N	N
MA: RoB impact of studies	1	N	N
RoB of studies in result interpret.	1	0	1
Result heterogeneity discussed	1	1	1
MA: publication bias analysis	0	N	N
Reported on conflict of interest	1	0	1
Total	14/16	1½/16	11/16

*Note.* 16 AMSTAR2 questions (answered through our extracted results). Yes = 1, Partial Yes = ½, No = 0, MA = Meta-analysis, N = No meta-analysis conducted.

### Study Quality and Risk of Bias

Quality assessment results for the five original studies are displayed in Table 2. Only one study scored as high-quality (Polenick et al., 2020). Quality assessment results for the systematic reviews are displayed in Table 3. For instance, as the original studies in reviews often had clinical health effects, we would apply the evaluation criteria to them, omitting adherence and other intermediate outcomes. The maximum was 16 points, of which three could be awarded for meta-analyses on original studies that all passed our inclusion criteria. We also scored the three meta-analysis questions through the extracted material. Only Cross et al. (2020) presented meta-analyses on dispenser devices. The importance of included studies and their results and their methodological quality vary—regardless of the review quality that gathered the evidence. For original studies of the earlier reviews, we used quality assessments found in the reviews. Low quality of RCTs, insufficient reporting of results, and heterogeneities in study outcomes prevented us from conducting meta-analyses. Instead, we used the above-defined rules to assess evidence levels.

### Outcomes of Dispenser Devices

In this section, we consider the results of this systematic review grouped by the Quintuple Aim domains:

1. Care experiences of older adults and caregivers
2. Well-being and experiences of care professionals (e.g., workload, job satisfaction)
3. Health and/or well-being outcomes
4. Use of health and/or social care/costs/cost-effectiveness
5. Equity

#### Care Experiences of Older Adults and Caregivers

##### Patient Satisfaction With Care

Two studies included in reviews report results on patients’ satisfaction with care. Valenstein et al. (2011) found no significant changes in patient satisfaction (Boeni et al., 2014—review included in Singh & Varshney, 2019). In the study by Henriksson et al. (2016), six participants prematurely withdrew from the electronic medication monitoring trial due to feeling overly monitored (Stevenson et al., 2019). One original study found an increase in patient needs median score due to automated medication dispensers (Hoffmann et al., 2018). Considering the (lack of) significance and consistency of the results, we conclude that there is no evidence of a beneficial effect of dispenser devices on patient satisfaction with care.

##### Caregiver Burden Outcomes

We found caregiver burden outcomes in three original studies (Hoffmann et al., 2018; Kamimura, 2019; Polenick et al., 2020). Introducing automated home dispensers appears to have a positive effect (Kamimura, 2019) or at least not to increase the care burden of caregivers; however, their tasks increased (Hoffmann et al., 2018). Polenick et al. (2020) compared automated and manual dispensers to usual care (no dispenser) and found that role overload was without dispensers higher for dementia caregivers than for caregivers caring for persons without dementia. Dispensers reduced the role overload of dementia caregivers significantly, forming limited evidence on it.

#### Well-Being and Experiences of Care Professionals

We found no studies where the effects of dispenser devices on care professionals’ experiences or well-being were assessed.

#### Health and/or Well-Being Outcomes

Most of the included studies report findings on various health outcomes, either self-reported or clinical outcomes. To ease the interpretation of the findings, we describe the results by study types by first considering findings from original studies and then from systematic reviews. Table 4 lists studies with significant results in the main subcategories of health outcomes, together with their numbers out of total number of studies in the categories.

**TABLE 4:** Main Results of Health Outcomes From Dispenser Devices

Outcome	Number of studies with significant findings out of total studies on outcome categories	
BP, cholesterol, HbA1C	5/8	Binstock & Franklin (1988), Simmons et al. (2000), Maier et al. (2006), Lee et al. (2006), Schneider et al. (2008)
Other clinical outcomes	4/6	Peterson et al. (1984), McPherson-Baker et al. (2000), Nochowitz et al. (2009), Mertens et al. (2020)
Symptoms	2/6	Peterson et al. (1984), Miaskowski et al. (2004)
Quality of life	3/7	Marek et al. (2013), Hale et al. (2016), Arain et al. (2021)

*Note*. Hale et al. (2016) Health-related quality of life result has nonbeneficial direction. Quality of Life category also includes anxiety, cognitive, and self-perceived health.

### Original Studies of Dispenser Devices

Four of the five original studies included reported on the effects of devices on health and well-being outcomes. Mertens et al. (2020) found that multidose drug-dispensing systems (prepackaged) with patients using vitamin K antagonists increased time in therapeutic range (TTR) significantly. In the same study, time under (TuTR), above (TaTR) therapeutic range, number of control visits, International Normalized Ratio (INR) assessments >4%, bleedings, and thromboembolic events were also included as outcome measures, but only the decreasing effect of the intervention on TuTR reached significance. Three studies explored the effects on quality of life (QoL). Arain et al. (2021) reported a significant self-rated health improvement, and Hoffmann et al. (2018) showed an increase in the median Montreal Cognitive Assessment score. Kamimura (2019) followed health outcomes of four patients over time and found that with the use of an automated dispenser device, the Clinical Dementia Rating–Global Score improved or stayed at least the same, whereas the Mini-Mental State Examination score, for which the device had mixed effects, mostly changed to the positive direction. We found limited evidence of beneficial effect of dispensers on self-rated health, TTR, and TuTR.

### Systematic Reviews on Dispenser Devices

Generally, systematic reviews report original study results directly. Only Singh and Varshney’s (2019) review contained results of another review (Boeni et al., 2014) that passed our inclusion criteria with its original study results on drug reminder packaging (RP), including multicompartment adherence (MCA) aid, multidrug punch (MDP) card, and unit-of-use packaging. When dispensing was done earlier and drugs were prepackaged, we classified packaging solutions as dispenser devices.

#### Blood Pressure, Cholesterol, and HbA1c

##### Blood pressure

Boeni et al. (2014) reported blood pressure (BP) results for included original studies. Rehder et al. (1980) found that RP—in the form of an MDP card—had no significant effect on BP unless it was part of a multiple intervention. Binstock and Franklin (1988) and Lee et al. (2006) presented significant decreases in systolic BP (SBP) and diastolic BP (DBP) due to RP. Schneider et al. (2008) also found a significant DBP decrease at 12 months follow-up due to using the MDP card, whereas the effects on SBP, absolute change in BP, and long-term outcome measures were not significant. Simmons et al. (2000) also found a significant DBP decrease from the use of the MDP card, but the SBP change was not significant. According to Boeni et al.’s (2014) assessment, only one of the above studies was a high-quality RCT (Lee et al., 2006). On the other hand, Cross et al. (2020) evaluated this same article as mediocre quality because participants and assessing pharmacists were not blinded to the dispenser device allocation. By our evidence levels, we find moderate evidence supporting the beneficial effect of dispenser devices on SBP and DBP levels.

##### Cholesterol

Lee et al. (2006) was reviewed by both Cross et al. (2020) and Boeni et al. (2014). Only the latter reported a temporary significant beneficial effect on low-density lipoprotein cholesterol levels due to the dispenser device (MDP card), presenting limited evidence of it.

##### HbA1C levels

Two RCT studies by Maier et al. (2006) and Simmons et al. (2000)—both in Boeni et al. (2014)—found significant reductions in HbA1C levels after exposure to the MDP card and MCA aid. According to Boeni et al. (2014), Maier et al. (2006) applied high-quality methodology. We found moderate evidence of beneficial effect of dispenser devices on HbA1C levels.

#### Other Clinical Outcomes

Boeni et al.’s (2014) systematic review reported significant effects of MCA aid on opportunistic infections with increased medication intake in McPherson-Baker et al. (2000), forming limited evidence on it. In the same review, Nochowitz et al. (2009) found significant effects of MCA aid for subtherapeutic INR values and time spent in therapeutic range, and Peterson et al. (1984) for patients within therapeutic range after exposure to MCA aid. Together with the earlier original Mertens et al. (2020) study’s result—with only moderate methodological quality in all three significant results—the above findings still constitute limited evidence of beneficial effect of dispensers on TTR.

#### Symptoms

Boeni et al.’s (2014) review reported significant beneficial effects of RP (MCA aid) on seizure frequency in the original study by Peterson et al. (1984) and average, worst, and least pain in the original study of Miaskowski et al. (2004). In Valenstein et al. (2011)—also in the Boeni et al. (2014) review—the change in psychiatric symptoms was not significant after exposure to the MDP card. Stevenson et al.’s (2019) review reported reductions in the number of rejection episodes in the original study by Henriksson et al. (2016) after exposure to electronic monitoring dispenser trays. On various symptoms, including rejection episodes, the direction of the effect of dispenser devices is generally beneficial, but reviews do not report significant findings. We found limited evidence of the beneficial effect of dispenser devices on symptoms.

#### Quality of Life

Hale et al. (2016)—reported in the Cross et al. (2020) review—found that remotely monitored electronic devices had a negative effect on the health-related QoL (HrQoL) but findings are likely to suffer from selection bias due to the differences in HrQoL between the treatment and the control groups at baseline. In the same review, Marek et al. (2013) found dispenser devices (both automated and nurse-filled) significantly improved both mental and physical HrQoL. The effects favored automated devices over nurse-filled ones, but the differences were not significant. Valenstein et al. (2011)—in the Boeni et al. (2014) review—studied the effects of RP on QoL but found no significant effects. According to our evidence levels, we found no evidence of the beneficial effect of dispenser devices on QoL. The significant results are inconsistent since the original studies by Arain et al. (2021) and Marek et al. (2013)—which favor intervention—are not sufficient to reach 75% of the total number of studies against the opposing result by Hale et al. (2016).

#### Service Utilization, Costs and Cost-Effectiveness

##### Service Use Outcomes

Five studies, of which four were included in systematic reviews, assessed service use outcomes. Stevenson et al.’s (2019) systematic review reported the beneficial effects of dispenser devices on unplanned admission rates to hospital or emergency department (ED) in Henriksson et al. (2016), but the results were not significant. McPherson-Baker et al. (2000) found a significant effect of MCA aid on hospitalizations, but Boeni et al.’s (2014) review considered this finding to be weak quality evidence. A meta-analysis conducted by Cross et al. (2020) on the results of Hale et al. (2016) and Winland-Brown and Valiante (2000) concluded that dispenser devices reduced the risk of ED and hospital readmissions significantly, but Cross et al. (2020) classified this evidence as weak quality. An original study by Mertens et al. (2020) found no significant change in hospital admissions due to robot prepackaged, multidose, drug-dispensing sachets with patients using vitamin K antagonists. Our evidence levels show limited evidence of beneficial effects on ED or hospital admissions.

##### Costs and Cost-Effectiveness

Three dispenser device studies reported in two reviews studied costs or cost-effectiveness. In the Stevenson et al. (2019) review, Henriksson et al. (2016) reported savings in the costs of unexpected treatments (such as rejections and unplanned hospital admissions). Boeni et al. (2014) reviewed reported findings from the two studies by Skaer et al. (1993a, 1993b) concentrating on diabetes and hypertension. Both studies concluded that the dispenser device (predispensed unit-of-use RP) yielded significant reductions in physician, hospital, and total expenditures. We found only limited evidence on costs and cost-effectiveness because Boeni et al. (2014) evaluated significant results by Skaer et al. (1993a, 1993b) to have low quality.

#### Equity

No study evaluated the effects of dispensers on equity.

### Subgroup Analyses

#### Research Method, Geographical and Patient Age Distributions

Studies were predominantly RCTs, which did not differ from the other study types in terms of result significance. We also found no apparent indication that significant findings would be more common in some geographical areas or more prevalent in certain age groups.

#### Age-Varying Effects of Dispensers and Statistical Estimates

Aging and age-varying effects of dispensers had only a minor role in the included studies. We found only one statistical estimate. For the HbA1C outcome, Maier et al. (2006)—included in Boeni et al. (2014)—reported that those younger than 55 benefited significantly more from a dispenser device compared to older adults. Older adults are often those with higher morbidity and more complex medication regimens, which makes it clearer that possibilities for activities outside the intervention (also implied by George et al., 2007) improved results for the younger adults since more benefits came with more medications to handle. Otherwise, results support dispenser devices for the older ones. The benefits of technologies that demand less from their users become more visible where age and medication management problems were greater.

## DISCUSSION

We included eight articles for data extraction and quality assessment that met our inclusion and exclusion criteria (five original studies and three reviews containing results on 20 additional original studies with older publication dates). In total, we extracted results from 25 original studies. Results from original studies generally favor interventions but empirical findings are often nonsignificant or in systematic reviews significance is not often reported. After quality assessment, we found moderate evidence of beneficial effects on SBP, DBP, and HbA1C levels. For other outcomes, no or only limited evidence was found.

On the Quintuple Aim domains, the following results were gathered. Most extracted studies report health effects (21 out of the 25). The effects of dispenser devices on health outcomes were significant in 13 of the 21 studies. Costs and cost-effectiveness results were found in three studies—two of which reported significant findings—but their evidence was classified as methodologically weak. Service use was assessed in five studies, and two of them reported significant results. We found one meta-analysis in Hale et al. (2016) and Winland-Brown and Valiante (2000), but Cross et al.’s (2020) review assesses them as weak in methodological quality. Patient satisfaction and caregiver burden were assessed in five studies. No study reported significant results on patient satisfaction, but a result for dementia caregivers indicated reduced role overload due to dispenser devices (Polenick et al. (2020). No study was found to evaluate professional well-being or equity, although more and more people have health or literacy constraints limiting possibilities for device-aided self-care.

Subgroup analysis showed no apparent differences in significant findings by methodology, geographical entity, or patient age, except for cases with limited research in some of their ranges. Dispenser research was from high-income countries only, focusing mainly on studies with mean patients ages 64–80. Intervention length has two concerns related to health outcomes. One is the medication loss of pharmacologic strength due to improper handling. A learning curve required for dispenser use may contribute to the relative success of interventions over longer periods, and run-in periods minimize its impact (Lee et al., 2006). Of the eight studies in which interventions lasted for 1 year, six reported significant findings (Binstock & Franklin, 1988; Marek et al., 2013; Mertens et al., 2020; Schneider et al., 2008; Skaer et al., 1993a, 1993b). Improved tolerance to increased medication use is another factor, but it should weaken observed effects compared to controls and reduce false positives.

The Quintuple Aim framework, as a basis, highlighted that the literature overlooks care professionals’ experiences and equity considerations, which are essential for informed recommendations to policymakers and care providers on the design and implementation of sustainable technological solutions. The literature did not distinguish between dispensing and reminding interventions, making systematic reviews challenging to perform and making recommendations very weak. Dispensers with higher technology than currently studied may improve QoL for older adults at their homes, alleviate the work pressure of care professionals and reduce service use, and control costs. High-quality research is needed on the effects of PMMDs on costs and service use. Evaluating access to these devices and subgroup analyses in terms of age, geographic area, limited health literacy, and education levels are also needed.

### Strengths and Limitations

We avoided multiple interventions and excluded adherence reports, focusing instead on health outcomes of the devices, which is a strength of our approach. This review also contributed to a more comprehensive systematic review format, including parallel screening with broader search terms, thus synthesizing information from various study types, including additional original study results and quality assessments established by earlier systematic reviews. A weakness was that some of the older data extracted from systematic reviews was not in the format for meaningful comparison and lacked reporting of statistical tests. Heterogeneities in outcome measures and interventions in terms of methodology, populations, and device types made it difficult to draw conclusions.

**Figure:**
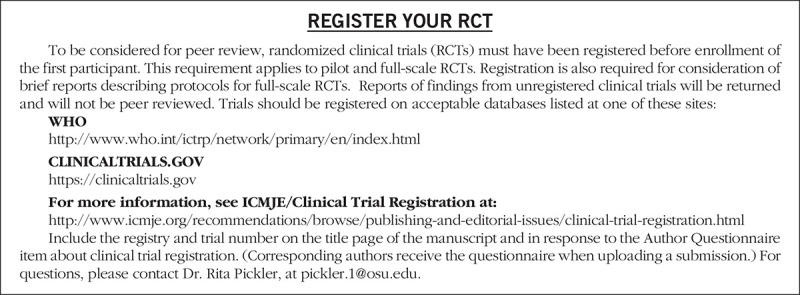


## CONCLUSION

We found moderate quality evidence to support health benefits in selected chronic disease outcomes for home-dwelling older adults, but no substantial evidence in any of the Quintuple Aim domains. The current literature does not yet support the conclusion that medication dispensers would be an effective measure to contain the growth of health and social care costs due to population aging. More high-quality research is needed to reach a definitive answer to this question.
